# Dual Drug Repurposing: The Example of Saracatinib

**DOI:** 10.3390/ijms25084565

**Published:** 2024-04-22

**Authors:** Raquel Ramos, Nuno Vale

**Affiliations:** 1 PerMed Research Group, Center for Health Technology and Services Research (CINTESIS), Rua Doutor Plácido da Costa, 4200-450 Porto, Portugal; raquel_ramos00@hotmail.com; 2 CINTESIS@RISE, Faculty of Medicine, University of Porto, Alameda Professor Hernâni Monteiro, 4200-319 Porto, Portugal; 3 Department of Community Medicine, Health Information and Decision (MEDCIDS), Faculty of Medicine, University of Porto, Rua Doutor Plácido da Costa, 4200-450 Porto, Portugal

**Keywords:** saracatinib, cancer, Alzheimer’s disease, drug repurposing, pre-clinical trials, clinical trials

## Abstract

Saracatinib (AZD0530) is a dual Src/Abl inhibitor initially developed by AstraZeneca for cancer treatment; however, data from 2006 to 2024 reveal that this drug has been tested not only for cancer treatment, but also for the treatment of other diseases. Despite the promising pre-clinical results and the tolerability shown in phase I trials, where a maximum tolerated dose of 175 mg was defined, phase II clinical data demonstrated a low therapeutic action against several cancers and an elevated rate of adverse effects. Recently, pre-clinical research aimed at reducing the toxic effects and enhancing the therapeutic performance of saracatinib using nanoparticles and different pharmacological combinations has shown promising results. Concomitantly, saracatinib was repurposed to treat Alzheimer’s disease, targeting Fyn. It showed great clinical results and required a lower daily dose than that defined for cancer treatment, 125 mg and 175 mg, respectively. In addition to Alzheimer’s disease, this Src inhibitor has also been studied in relation to other health conditions such as pulmonary and liver fibrosis and even for analgesic and anti-allergic functions. Although saracatinib is still not approved by the Food and Drug Administration (FDA), the large number of alternative uses for saracatinib and the elevated number of pre-clinical and clinical trials performed suggest the huge potential of this drug for the treatment of different kinds of diseases.

## 1. Saracatinib: An Overview

Saracatinib (AZD0530), N-(5-chloro-1,3-benzodioxol-4-yl)-7-[2-(4-methylpiperazin-1-yl)ethoxy]-5-(tetrahydro-2H-pyran-4-yloxy) quinazolin-4-amine, is an orally available, and highly selective dual-specific c-Src/Abl kinase inhibitor developed by AstraZeneca with activity in the nanomolar range, inhibiting Src (v-src avian sarcoma (Schmidt-Ruppin A-2) viral oncogene homolog) and Abl (Abelson murine leukemia viral oncogene homolog) with an IC_50_ of 2.7 and 30 nM, respectively [1,2,3]. Two types of dual Src/Abl inhibitors exist, type I and II. Type I inhibitors target the ATP-biding site in the active conformation. Otherwise, type II inhibitors were first described for Abl and targeted the ATP-binding site; however, they can perform their action in the protein-closed conformation. Additionally, these type II inhibitors are also capable of occupying an adjacent hydrophobic pocket [3]. Considering the binding characteristics of saracatinib that will be further explained, it can be classified as a type II Src/Abl inhibitor [4]. This drug was initially studied for the treatment of solid tumours, alone or in combination with other chemotherapeutic agents, in advanced and metastatic stages, targeting Src, an SRC family of tyrosine kinase (SFKs) member. Pre-clinical studies demonstrated promising anticancer effects in several types of cancers. In the clinical setting, phase I clinical trials also demonstrated encouraging results about safety and drug tolerability. Consequently, several phase II clinical trials were also performed [3]. In addition to those conducted for cancer, pre-clinical and clinical studies were performed to evaluate the efficacy of saracatinib in Alzheimer’s disease (AD) treatment, considering the association between Fyn and the cascade Aβ-PRP^c^ [5,6]. Several in vivo assays showed promising results for the use of saracatinib in Alzheimer’s treatment, evidencing the reversal of memory deficits in a transgenic mouse model, rescued synapse density, suppression of Tau aggregation, and normalisation of hippocampal synaptic density. Moreover, saracatinib was shown to sustain the previously described effects even after drug washout [6,7,8,9]. Concomitantly, phase I (NCT01864655) and phase II (NCT02167256) clinical trials for the use of saracatinib in AD were also performed, proving that saracatinib is safe and well tolerated by mild-to-moderate AD patients, and is capable of penetrating the human blood-brain barrier (BBB) and achieving cerebrospinal fluid (CSF) with oral dosing between 100 and 125 mg [10,11]. 

**Figure 1:**
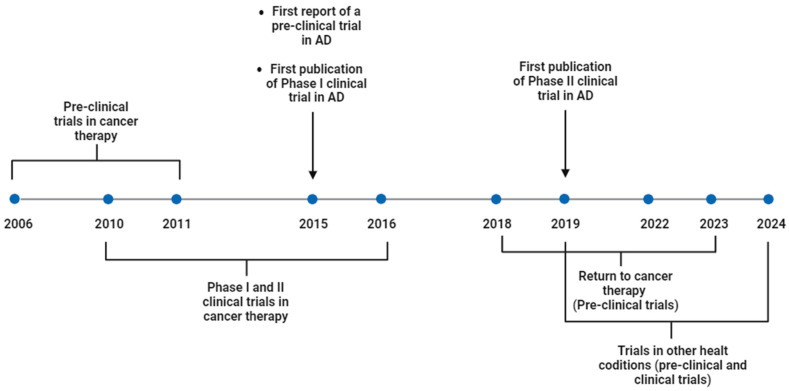
Timeline of pre-clinical and clinical trials testing saracatinib for the treatment of different health conditions. Created with BioRender.com. Available online: http://biorender.com/ (accessed on 28 February 2024).

Currently, despite all these promising results, saracatinib has not yet been approved by the Food and Drug Administration (FDA). However, studies testing this drug for the treatment of different diseases and health conditions are continually emerging, reflecting the huge interest in this drug for clinical use. Therefore, with this article, we aim to review all the stages that saracatinib has been through, highlighting the importance of drug repurposing. Figure 1 shows a summing up of the history of this drug that will be detailed in this article. 

### 1.1. Pharmacodynamics

#### 1.1.1. Drug-Target Interaction

The kinase domain of the SFKs has a high sequence conservation which leads to a limited selectivity of Src inhibitor [12]. Although the objective of most Src/Abl inhibitors is to compete with ATP on the active site or decrease the open and active protein conformation, careful studies on the target structure and the ligand–protein interaction are crucial for a well-designed inhibitor [2,4,13]. Therefore, to maximise the interaction between the saracatinib and the target, studies were performed to optimise the drug structure and increase the protein inhibition [2,14]. Later a study with recurrence to X-ray crystallography to analyse the interaction between saracatinib and Src demonstrated that the inhibitor is capable of binding to the inactive kinase conformation, instigating a change in the catalytic site of ATP and allowing the hydrophobic pocket to become available for drug binding. The mechanism of inhibition is identical in Abl [3,4].

#### 1.1.2. Adverse Events

As will be explained, saracatinib appeared to be well-tolerated at once-daily doses up to 175 mg in phase I trials for cancer therapy; however, some secondary events were also described. Some dose-limiting toxicities included cytopenia, asthenia, and pulmonary toxicity. Other adverse effects were the increased aspartate aminotransferase and alanine transaminase levels, nausea, anorexia, myalgias, cough, neutropenia, and thrombocytopenia. Time-dependent P450 enzyme inhibition was another effect [15,16].

These adverse events may be related to the fact that saracatinib is a 1,3-benzodioxole group-containing compound. These compounds were described to induce P450-dependent toxicities, once P450-mediated metabolic activation begins with a demethylation of 1,3-benzodioxole leading to catechol production. Subsequently, these are oxidised to ortho-quinones which are highly reactive and have a cytotoxic effect owing to the production of reactive oxygen species (ROS) [15,17,18].

### 1.2. Pharmacokinetics (PK)

The metabolism of saracatinib is mainly performed by cytochrome P450 3A4 (CYP3A4), and involves metabolism by N-demethylation to M594347, and possibly leads to secondary effects, namely gastrointestinal disorders, by the mechanism previously described [19,20]. In vitro studies demonstrated the saracatinib capability of high distribution into tissues, and complementary to this, in vivo findings demonstrated its good PK characteristics, which means it has good aqueous solubility and moderate binding to plasma proteins [21,22].

In humans, PK characteristics can change considering the differences among population metabolisms, which vary according to ethnicities, for example [20]. Overall, the two clinical studies on Caucasian and Japanese patients demonstrated that saracatinib in monotherapy is well tolerated by both populations. Nevertheless, the maximum tolerated dose (MTD) for cancer treatment in the Japanese population is lower than that defined for Caucasians (125 mg vs. 175 mg). However, despite the MTDs being different, the PK parameters remain substantially similar, with a half-life (t_1/2_) of approximately 40 h that allows daily dosing (Table 1). These studies also confirmed the excellent oral availability of saracatinib (>90%) [19,20]. Moreover, the PK of saracatinib in combination with carboplatin and/or paclitaxel was also studied in patients with advanced solid tumours and the PK parameters described were similar to the ones seen with saracatinib monotherapy [23]. The combination of saracatinib with cediranib (an oral inhibitor of vascular endothelial growth factor (VEGF) signalling) did not show either pharmacokinetic interaction [24].

## 2. Src as a Molecular Target

SFKs are composed of nine members with a similar structure: c-Src (Src), LYN, FYN, LCK, HCK, FGR, BLK, YRK, and YES. They can be activated by receptor tyrosine kinases (RTKs), are involved in the activation of several signalling pathways, and participate in multiple cellular processes such as proliferation, survival, adhesion, and migration [1,25].

Structurally, SFKs proteins can appear in both active and inactive conformations depending on the phosphorylation of negative regulatory tyrosine residue (Tyr530) and the interaction with the SH domains—SH1 (catalytic domain and ATP binding site), SH2, and SH3 [1,3,21]. Specifically, the Tyr530 phosphorylation induces an intramolecular association between this residue and the SH2 domain of Src, leading to a closed and inactive protein conformation. This conformation is stabilised by an interaction between the SH3 domain and a proline-rich stretch. Conversely, the dephosphorylation of Tyr530 and autophosphorylation of Tyr419 in the catalytic domain leads to an open and active form of Src [1].

c-Src was the first protein kinase to be described as capable of phosphorylating tyrosine residues [26], being the most well researched member of the SFKs family and the most frequently involved in carcinogenesis [27]. The *c-Src* proto-oncogene was discovered in 1976, and later, in 1980, Src was described as a protein tyrosine kinase. In subsequent years, the Src homology domains were identified and in 1999 the first *Src* mutations were described in colorectal cancer [28]. As previously mentioned, this gene encodes a nonreceptor tyrosine kinase involved in several cellular processes, namely proliferation, survival, migration, and angiogenesis. Most cell types have low expression levels of Src and, despite only being activated during the cellular processes in normal cells, its high expression and activation are related to cancer progression and metastases [1]. Moreover, studies on cancer cells also showed that Src is involved with PI3K/Akt/mTOR, MAPK, and PDGF signalling pathways as well with signal transducers and activators of transcription (STATs), important factors for carcinogenesis [25]. Concomitantly, Src also plays an important role in osteoclast activation and bone resorption. However, it can end in a bone metastases scenario in case of aberrant expression [22].

On the other hand, Bcr-Abl is encoded by the Philadelphia chromosome, being the etiologic agent of chronic myeloid leukemia (CML). c-ABL, the cellular homolog of Bcr-Abl, is involved in several cellular processes, promoting multiple transduction cascades, the growth, proliferation, and survival of hematopoietic cells, inhibition of apoptosis, and alteration of cellular adhesion [22]. Bcr-Abl is the target for CML treatment and, as SFKs family members, it can also exist in an active or inactive conformation. Here the SH2 and SH3 domains are essential for regulated Abl activity, and its activation is performed by the phosphorylation of some residues, such as Tyr-412 and Tyr-245. This protein is capable of inhibiting apoptosis, inducing cell proliferation, and activating other nonreceptor TKs, such as SFKs, namely Hck and Lyn [3,29].

Therefore, owing to the involvement of Src in cancer progression, the development of molecular inhibitors of SFKs became important. Moreover, although the administration of imatinib is the first therapeutic approach for CML, the development of other molecular inhibitors is crucial due to the development of drug resistance and for more advanced phases of disease [1,3]. Taken together, Src and Abl have a high structural homology (identity of 47% in the catalytic domains of human Src and Abl), and, consequently, many compounds that were initially designed as Src inhibitors were also shown to be active against Abl. Saracatinib is one of these dual Src/Abl inhibitors and other examples are bosutinib and dasatinib, with this last one already approved for the treatment of imatinib-resistance CML in the USA and Europe [3].

## 3. Evolution in Cancer

As mentioned before, Src is involved in several human cancers, such as colorectal cancer, breast cancer, lung cancer, pancreatic cancer, gastric cancer, ovarian cancer, bladder cancer, head and neck cancer, brain cancer, melanoma, and leukemias/lymphomas. Thus, the therapeutic effect of saracatinib in monotherapy or combined with other drugs has been studied in pre-clinical and clinical trials [16].

### 3.1. Pre-Clinical Studies

The antitumour effects of saracatinib alone or in combination, have been observed in some cancer cell lines, namely breast, prostate, and lung, since around the year 2006 [16]. In breast cancer cell lines, treatment with saracatinib and tamoxifen was shown to be effective in the prevention of acquired antihormone resistance and in the reduction in protein levels involved in tumour progression. Concomitantly, in tamoxifen-resistant breast cancer cell lines, the combined therapy of saracatinib with gefitinib, an EGFR inhibitor, resulted in a higher cell adhesion and a reduction in the cell-invasion capability. The results for prostate cancer cell lines were also promising, showing a reduction in cell mobility [16,30,31]. The induction of apoptosis, inhibition of tumour growth and cell invasion, and cell cycle arrest were other promising results obtained after Src inhibition with saracatinib in cell lines of head and neck squamous cell carcinoma, lymphoma, and colorectal cancer [32,33,34]. In addition, in lung cancer cell lines, in addition to the inhibition of tumour cell invasion, treatment with saracatinib has shown to increase radiosensitivity [35].

### 3.2. Clinical Studies

Considering the promising results from pre-clinical studies, phase I and II clinical trials were performed in order to determine the safety of saracatinib and the patients’ tolerability to the drug in monotherapy or in combination. Phase I clinical studies confirm the oral availability of saracatinib and reveal that this drug is well tolerated by patients at doses up to 175/125 mg, demonstrating a successful reduction in tumour Src activity. Therefore, considering the tolerated doses defined in the studies, a recommended daily dose of 175 mg and 125 mg of AZD0530 for European and Japanese patients, respectively, was proposed. These doses were then taken into consideration for phase II clinical trials [19,20]. In addition, another phase I trial to evaluate the impact of saracatinib in bone metastases showed great results, suggesting that this Src inhibitor may have a therapeutic effect in metastatic bone disease [36]. Combined therapy with paclitaxel and/or carboplatin also demonstrated an acceptable toxicity [23]. A treatment combination of cediranib, an oral inhibitor of VEGF signalling, with saracatinib was also shown to be efficient and well tolerated by patients with advanced solid tumours at doses of 175 mg/day of saracatinib plus 20 or 30 mg/day of anti-VEGF [24].

Despite these encouraging phase I results, phase II studies showed that saracatinib had limited therapeutic efficacy in monotherapy or combined therapy, and several adverse events occurred in the majority of cancers [16]. Curiously, the phase II trial in platinum-pretreated Non-small cell lung cancer (NSCLC) patients from 2014 was the only trial that showed positive results with minimal side effects, partial therapeutic response, and tumour stabilisation. In addition, this trial also demonstrated that there probably exists a subset of saracatinib-responsive NSCLC that was not defined in the study [37]. Concordantly with these results, a study from 2015 using pre-clinical models also demonstrated the therapeutic action of saracatinib in a subset of NSCLC, depending on the *EGFR*/*RAS* mutational profile. Specifically, they showed that cells carrying the *EGFR* T790M mutation and with erlotinib-resistance are more responsive to the combined treatment with saracatinib and cetuximab. Conversely, models with mutated *RAS* and resistance to erlotinib showed a better response to dasatinib in combination with the *MEK* inhibitor selumetinib [38].

To the best of our knowledge, the clinical trials of saracatinib for cancer treatment stopped in 2016, with no articles published since then. Nevertheless, in the last year, a randomised phase II clinical trial testing the therapeutic efficacy of an aromatase inhibitor combined with saracatinib in hormone receptor-positive metastatic breast cancer was published after promising results in a phase I trial [39,40]. This study was based on pre-clinical data that showed the efficacy of saracatinib in enhancing the anti-proliferative effects of endocrine agents in breast cancer cell lines [41]. Moreover, bone is the most common site of metastases in hormone-sensitive breast cancer, and considering the capability of saracatinib to inhibit osteoclast activity, this area was also evaluated in the trial [36]. Unfortunately, the trial revealed that saracatinib was incapable of improving the patients’ outcomes under the study conditions, and showed no promising effect on bone metastases either [39]. However, despite this, the article seems to recommend a return to saracatinib for cancer treatment, and a clinical trial was conducted between 2012 and 2015. Therefore, no efforts to reduce saracatinib toxicity were made and the side effects were similar to those reported in other clinical trials. The same situation was observed in a clinical trial published in 2020, which aimed to determine if saracatinib is capable of increasing progression-free survival (PFS) in patients with a complete resection of osteosarcoma lung metastases. This trial was performed on patients between 2009 and 2014, following a therapeutic scheme implemented in 2012 [42]. Table 2 summarises the cancer types submitted into phase II trials.

### 3.3. Resistance in Cancer

Considering the generally unpromising results obtained in phase II clinical trials, more recent studies were aimed at justifying the reason for the low anti-tumour activity of saracatinib, one of the possible causes being the development of mechanisms of resistance. Specifically, one pre-clinical study was aimed at understanding why saracatinib did not improve the outcomes of women with platinum-resistant ovarian cancer, and their results suggest that the activation of the MAPK signalling pathway through the reduction in NF1 (neurofibromin) or overexpression of HER2/insulin receptor leads to resistance to AZD0530 in ovarian cancer cell lines. Concomitantly, a combination of Src inhibition and MEK inhibitor showed a synergistic effect in platinum-sensitive and resistant ovarian cell lines [54]. On the other hand, microRNAs (miRNAs) also seem to be important in saracatinib’s low therapeutic action, once their dysregulation is highly associated with resistance to anti-cancer drugs in several cancers [55]. Therefore, a study in breast cancer cells revealed that the downregulation of miR-19b-3p increases the IC_50_ value of saracatinib, promoting cell proliferation and saracatinib resistance. Moreover, it showed that this miRNA is related to PIK3CA expression, and its downregulation leads to the activation of the PI3K/AKT signalling pathway, resulting in drug resistance. Therefore, future pre-clinical and clinical studies testing the dual inhibition of Src and PI3K could be advantageous [56].

## 4. Repurposing Saracatinib in Alzheimer’s Disease

As is already known, saracatinib was first designed for cancer treatment, due to the involvement of Src with tumourigenesis. However, saracatinib was never approved by the FDA for cancer therapy due to the toxicity observed in phase II clinical trials. Therefore, the interest of saracatinib in AD has increased over the years due to the association of Fyn, another SFKs family member, with important proteins related to AD (Aβ and Tau). The first association between Fyn and AD was made in 1993 when a research group described a strong Fyn immunoreactivity in neurons from an AD brain; the same neurons also have an abnormal phosphorylation of the Tau protein [57]. Consequently, the use of saracatinib to inhibit Fyn and help in the treatment of central nervous system (CNS) diseases, such as Alzheimer’s disease, seems promising [13,25].

### 4.1. Fyn as a Molecular Target

Specifically, Fyn is another member of SFKs family involved in some biological functions, such as T-cell receptor signalling, cell division and adhesion, synaptic function and plasticity, and central nervous system myelination [57]. This protein was described as the phosphorylated microtubule-associated protein Tau, which accumulates in the brain leading to neurological conditions, such as Alzheimer’s disease. Deposition of amyloid-beta (Aβ) plaques is another Alzheimer’s-associated event and Fyn is also involved through the transmission of downstream signals triggered by the binding of Aβ oligomer to cellular prion protein (PRP^c^) with high affinity on the neuronal cell surface. This cascade can also lead to Tau phosphorylation [8,58,59]. Regarding the therapeutic option, several efforts were made to create drugs that work against amyloidopathy and the Tau protein; however, they failed [59]. One of the reasons for the failure of this therapeutic approach was the high proportion of side effects affecting essential biological functions when trying to reduce Aβ levels. Consequently, targeting Fyn kinase with saracatinib appears to be a promising therapeutic strategy for Alzheimer’s treatment. Additionally, although there are other members of the SFKs family expressed in the brain, only Fyn was shown to be activated by Aβ in vitro [57,60].

### 4.2. Dosage, Symptoms, and Pharmacokinetics

In Alzheimer’s disease, phase I clinical trials have demonstrated that the doses of saracatinib needed for therapeutic action and that are well tolerated by patients range from 50 mg to 125 mg once daily, which is lower than the doses required for cancer treatment. Nevertheless, some secondary effects such as diarrhea, headache, fatigue, and nausea, were described at higher doses of AZD0530 [10].

Considering the PK parameters, in vivo studies have revealed that the half-time of saracatinib in the brain is 16 h with a plasma:cerebrospinal fluid ratio of 3:1. Moreover, in phase I clinical trials, only doses of 100 mg and 125 mg have shown to be capable of crossing the BBB and achieving CSF levels that produced significant results in vivo (2.5 to 14.0 nM compared to 5.8 to 14 nM in mouse models) [6,10].

## 5. Other Repurposing Areas

In addition to the repurposing of saracatinib for AD treatment, the drug is also being studied for use against other health conditions because different cellular targets can also be inhibited by this drug. One study showed that Src also plays a role in myofibroblast differentiation and fibrogenic gene expression. Myofibroblast differentiation is a complex process that requires several factors, namely TGF-β, integrin signalling, and an adequate extracellular matrix. Consequently, despite this process being involved in wound healing, it also promotes the development of fibrotic disease in different organs. Therefore, the involvement of Src in this condition makes it a good target for saracatinib [61]. On the other hand, small molecule kinase inhibitors were also described as potential inhibitors of receptor kinase ALK2, which is involved in fibrodysplasia ossificans progressiva (FOP). Accordingly, since saracatinib is part of this group of drugs, its reuse in FOP therapy seems promising [62,63]. The Fyn-PKCδ signalling pathway is another cellular target under study for the application of saracatinib in the treatment of temporal lobe epilepsy (TLE). Previously, this signalling pathway was associated with neuroinflammatory mechanisms, leading to the production of ROS and proinflammatory cytokines, characteristics found in rat models of TLE [64,65]. Also, the necroptosis process appears to be a possible target for saracatinib, since this process is involved in the pathology of multiple diseases, such as bowel inflammation, neurodegenerative diseases, and autoimmune diseases. The mixed lineage kinase domain-like protein (MLKL) is the downstream mediator of the necroptosis process and also the molecular target for saracatinib that has shown promising results in the treatment of psoriasis, an autoimmune disease [66,67]. Additionally, the association of Src with the N-methyl-D-aspartate (NMDA) glutamate receptors, an important mediator of chronic pain hypersensitivity, with the activation of mast cells mediated by immunoglobulin E (IgE) makes saracatinib an interesting possible analgesic and anti-allergic drug, respectively [68,69].

The emergence of all these new cellular targets for saracatinib is an example of the high applicability of drug repurposing, a powerful method to accelerate the development and approval of new drugs for different diseases [70]. Table 3 summarises all the health conditions described above where saracatinib is used as a repurposed drug, elucidating the cellular target(s) and the trials that it has already been through in each disease/condition. While most of these are preliminary studies, further research is required to conclusively determine the drug’s efficacy. However, the investigations cited showed promising results in different areas, further demonstrating the high potential of saracatinib in clinical evolution. 

## 6. Saracatinib Returns to Cancer Treatment

As the anti-cancer efficacy observed in phase II trials did not show promise for further studies, no additional clinical trials testing different approaches to achieve therapeutic success with saracatinib have been performed and published since 2016. However, some pre-clinical trials arose after that time, aimed at developing novel saracatinib-based strategies for cancer treatment. One of the most studied strategies involves the use of nanoparticles (NPs) which have shown promising results in drug delivery considering their tumour-specific characteristics. Therefore, in 2018, a pre-clinical trial attempted to synthesise nanoparticles for the selective release of saracatinib in Head and neck squamous cell carcinoma (HNSCC). In vivo results showed an elevated anti-tumour efficacy of the NPs loaded with saracatinib (10 mg/kg) when compared to the free drug (20 mg/kg) [78]. Consequently, in the next year, once AKT was described by the authors as a cause of resistance to saracatinib, dual drug-loaded nanoparticles (NPs) to co-deliver saracatinib and capivasertib, an AKT inhibitor, were developed and tested in HNSCC. The results suggested that blocking AKT improves the therapeutic action of saracatinib. Moreover, the co-delivery of capivasertib and saracatinib by tumour-targeting NPs (10 mg/kg, ratio 1:1) appeared to achieve better treatment outcomes than the free drug combination. Importantly, this was accomplished without increasing side effects due to the highly specific tumour-targeting drug delivery system [79].

Pre-clinical trials from 2018, 2019, and 2023 testing new formulations with saracatinib in clear-cell renal carcinoma (ccRCCs) [80], glioblastoma [81], and castration-resistant prostate cancer (CRPC) [82], respectively, were also performed. Specifically, combined therapy with saracatinib and GDC-0941 (an inhibitor of PI3K) inhibits cell growth, and invasion, and promotes cell death in renal tumour cell lines [80]. For glioblastoma, in vitro and in vivo trials demonstrated that a combination of Lentivirus vectors containing siRNA-targeted STAT3 (LV-STAT3 siRNA) and AZD0530 has a synergistic effect, inhibiting proliferation and inducing the apoptosis of glioblastoma cells and tumour size reduction [81]. Encouraging in vitro results were also produced in the pre-clinical trial performed in CRPC cells, where the combined therapy of saracatinib and enzalutamide (a second-generation hormonal drug given to men with CRPR) resulted in a reduction in DNA replication and the subsequent increase of apoptosis in a subset of CRPC cells, including those positive for androgen receptor-full length and androgen receptor splice variants (AR-FL+/AR-V+) [82].

Naturally, regardless of the promising results, clinical trials provide the final answer regarding the safety, tolerability, and efficacy of this novel saracatinib delivery approach and the new combinatory drug treatments. However, to the best of our knowledge, no results from these trials have been published or presented to date. Consequently, while the initial pre-clinical trials were successful, the failure at the clinical trial level means that the efficacy of these recent saracatinib-based cancer treatments is still unclear.

## 7. Conclusions

Saracatinib (AZD0530) was first designed by AstraZeneca for use against cancer, and showed good PK parameters and a MTD of 125 mg and 175 mg in Japanese and Caucasians, respectively, according to the phase I trials. Unfortunately, the drug exhibited low therapeutic effects and high toxicity in phase II clinical trials in various types of cancer, reducing interest in its application to cancer treatment. However, the exception observed in NSCLC, where saracatinib showed promising results, leads us to believe that its efficacy is strictly related to the specific subtype of cancer. This highlights the need for specific biomarkers to accurately select the most suitable patients to receive this drug, enhancing therapeutic outcomes. In addition, the new pre-clinical studies that have recently arisen aimed at reapplying saracatinib in cancer therapy using nanoparticles or new pharmacological combinations seem to indicate interesting potential approaches to reduce drug toxicity and improve AZD0530 therapeutic efficacy. Besides cancer, the Src inhibitor continues to be studied in relation to other health conditions over time, with Alzheimer’s disease appearing as one of the most promising targets. Despite saracatinib remaining unapproved by the FDA, its repurposing in other diseases and health conditions reveals the interest in Src inhibitors for therapy. Thus, the overall path of saracatinib appears to involve a form of re-repurposing, backing cancer research after years without being studied in the context of this disease. This, allied to the other application of saracatinib under investigation, reinforces its potential as a therapeutic approach either in monotherapy or in combination with other drugs. 

## Figures and Tables

**Table 1:** Pharmacokinetics parameters for saracatinib in monotherapy in Japanese and Caucasian populations, considering the maximum tolerated dose (MTD) for each, after administration of a single dose [19,20].

	Japanese (125 mg)	Caucasian (175 mg)
C_max_ (ng/mL)	140	149
t_max_ (h)	4	2
t_1/2_ (ng/mL)	40	39

C_max_—maximum plasma drug concentration after single-dose administration; t_max_—time to reach maximal concentration following saracatinib administration; t_1/2_—time for saracatinib plasma concentration to decrease by 50%.

**Table 2:** Phase II clinical trials performed for various types of cancer. Identification by the ClinicalTrials.gov Identifier is represented.

Cancer Type	Monotherapy or Combined Treatment	Clinicaltrials.gov (accessed on 1 March 2024) Identifier	Ref
Metastatic melanoma	Monotherapy	NCT00669019	[43]
Hormone-receptor negative metastatic breast cancer	Monotherapy	NCT00559507	[44]
Hormone receptor-positive metastatic breast cancer	Combined with anastrazole (AI)	NCT01216176	[39]
Advanced gastric adenocarcinoma	Monotherapy (≤1 prior line of chemotherapy)	NCT00607594	[45]
Metastatic HNSCC	Monotherapy	NCT00513435	[46]
Advanced pancreatic cancer	Combined with gemcitabine	NCT00265876	[47]
mCRC	Monotherapy (one prior chemotherapy regimen)	NCT00397878	[48]
Advanced castration-resistant prostate cancer	Monotherapy (<1 prior taxane-based chemotherapy regimen)	NCT00513071	[49]
Platinum-resistant ovarian cancer	Combined with paclitaxel	NCT01196741	[50]
Thymic malignancy	Monotherapy	NCT00718809	[51]
Metastatic clear-cell renal cancer	Combined with cedirabin (after ≥1 VEGF-targeted therapy)	NCT00942877	[52]
SCLC	Monotherapy (platinum-pretreated)	NCT00528645	[53]
NSCLC	Monotherapy (platinum-pretreated)	NCT00638937	[37]
Recurrent osteosarcoma	Monotherapy	NCT00752206	[42]

HNSCC—Head and neck squamous cell carcinoma; AI—aromatase inhibitors; mCRC—metastatic colorectal cancer; SCLC—small-cell lung cancer; NSCLC—non-small-cell lung cancer.

**Table 3:** List of conditions where the efficacy of saracatinib as a repurposed drug has been studied. Clinical trials are identified by the ClinicalTrials.gov Identifier (accessed on 1 March 2024).

Condition	Cellular Target(s)	Model	Clinicaltrials.gov Identifier	Ref
Pulmonary fibrosis	Fibrogenic pathways (EMT, TGF-β, and WNT signalling)	In vitro/In vivo/Ex vivo	-	[71]
	Src	In vitro/In vivo	A clinical trial is already ongoing (NCT04598919)	[72]
Liver fibrosis	Fyn	In vitro/In vivo	-	[73]
FOP	ALK2/ACVR1-kinase	In vitro/In vivo	NCT04307953	[74,75]
TLE	Fyn-PKCδ signalling pathway	In vivo	-	[65]
Psoriasis	Necroptosis inhibition by targeting MLKL	In vitro/In vivo	-	[67]
Analgesic for cancer-induced bone pain	NMDA glutamate receptors	-	NCT02085603	[76]
Anti-allergic	Signalling cascades involved in allergic reactions *	In vitro/In vivo	-	[77]

FOP—Fibrodysplasia ossificans progressiva; TLE—temporal lobe epilepsy; ACVR1—bone morphogenetic protein (BMP) receptor kinase ALK2; EMT—epithelial-mesenchymal transition; MLKL—mixed lineage kinase domain-like protein; NMDA—N-methyl-D-aspartate. * Lyn, Akt, a PI3K substrate, and MAPKs.

## Data Availability

Not applicable.
